# *Boletus edulis* Extract—A New Modulator of Dysbiotic Microbiota

**DOI:** 10.3390/life13071481

**Published:** 2023-06-30

**Authors:** Ionela Avram, Diana Pelinescu, Florentina Gatea, Robertina Ionescu, Alexandru Barcan, Razvan Rosca, Anca Zanfirescu, Emanuel Vamanu

**Affiliations:** 1 Department of Genetics, University of Bucharest, 36-46 Bd. M. Kogalniceanu, 5th District, 050107 Bucharest, Romania; 2 Centre of Bioanalysis, National Institute for Biological Sciences, 296 Spl. Independentei, 060031 Bucharest, Romania; 3 Faculty of Biotechnology, University of Agricultural Sciences and Veterinary Medicine, 011464 Bucharest, Romania; 4 Anoom Laboratories SRL, București, 28 Vintila Mihaileanu Sector 1, 024023 Bucharest, Romania; 5 Faculty of Pharmacy, “Carol Davila” University of Medicine and Pharmacy, Traian Vuia 6, 020956 Bucharest, Romania

**Keywords:** pattern, porcini, functional product, in vitro, short-chain fatty acid synthesis

## Abstract

The regular administration of antibiotics is a public concern due to the prejudices of large population groups and the high frequency with which antimicrobial products are prescribed. The current study aimed to evaluate the in vitro effect of a new extract from *Boletus edulis* (BEE) on the human microbiota. One of the disadvantages of this extensive use is the disruption of the human microbiota, leading to potential negative health consequences. The in vitro evaluation of BEE consisted in determining its cytotoxicity, influence on the concentration of four types of cytokines (IL-6, IL-10, IL-1β, TNFα), and capacity to modulate the human microbiota after administering antibiotics. The latter was assessed by microbiome analysis and the evaluation of short-chain fatty acid synthesis (SCFAs). Simultaneously, the content of total polyphenols, the antioxidant capacity, and the compositional analysis of the extract (individual polyphenols composition) were determined. The results showed that BEE modulates the microbial pattern and reduces inflammatory progression. The data demonstrated antioxidant properties correlated with the increase in synthesizing some biomarkers, such as SCFAs, which mitigated antibiotic-induced dysbiosis without using probiotic products.

## 1. Introduction

Recent studies have proved the essential role of gut microbiota in human health, including its involvement in metabolizing nutritional compounds, maintaining a healthy digestive tract, and regulating the homeostasis of the immune system [1]. If this network is disrupted, pathogenic disorders and inflammation might develop. While antibiotics have been a worldwide game-changer in treating contagious illnesses, their use can have unintended consequences, hindering the development of healthy bacteria and impacting microbial strains within the digestive tract [2]. The reduction of gut microbiome diversity under antibiotic treatment directly impacts the interactions between the host and microbes, the immune system’s balance, and the host’s protection against harmful microorganisms [3].

Furthermore, gut microbiota may alter the effectiveness of specific medicinal treatments [4]. The edible mushroom *Boletus edulis* (porcini) has nutritional and medicinal value. The nutritional value resides in the high content of *carbohydrates*, proteins, minerals, and flavor components, while being low in calories and fat [5]. The medicinal qualities are linked to their content of bioactive substances, such as polysaccharides [6], which act by modulating gut microbiota. This indicates that, in the digestive tract, the polysaccharides function as prebiotics [7]. Prebiotics are dietary elements that undergo selective fermentation, leading to changes in the composition and activity of the microbiota in the gastrointestinal tract [8]. These changes provide the host with nutritional and health advantages. The majority of polysaccharides are found in *Boletus edulis* are β-glucan polymers, with the main chain consisting of β-(1→3) and β-(1→6) bounds. Furthermore, compounds such as chitin, mannans, galactans, and xylans are among the polysaccharides in *Boletus edulis* [9]. Because the digestive enzymes released by the stomach and pancreas cannot hydrolyze the β-glucosidic link, the non-digestible carbohydrates found in mushrooms can function as prebiotics in the digestive system.

Recent research in our lab demonstrated that two prebiotic compounds, RoBioMush1 and RoBioMush2, can potentially modulate the microbiota associated with cardiovascular disorders [10,11]. We aimed to verify if the extract of *Boletus edulis* could also counteract several adverse effects of antibiotics on the gut microbiota, such as decreased biodiversity, and distorted metabolic activity. To this purpose, we set out to do the following: (a) describe the extraction method of the functional product from *Boletus edulis*; (b) outline the cytotoxic effect and the immune response of *B. edulis* extract; (c) measure the polyphenolic compounds in samples by capillary zonal electrophoresis; (d) use the single-chamber system (GIS1) for in vitro simulation of the human gastrointestinal tract to test the effects of porcini mushroom extracts on dysbiotic microbiota after antibiotic consumption.

## 2. Materials and Methods

### 2.1. Substrate and Extraction Process

*Boletus edulis* was purchased through SC Anoom Biolaboratories SRL, from Calufunghi Mario SRL in Gorj County, Romania. The dried mushrooms were sorted and crushed with a universal mill to a uniform consistency. The resulting powder was stored in self-sealing bags.

**Figure 1:**
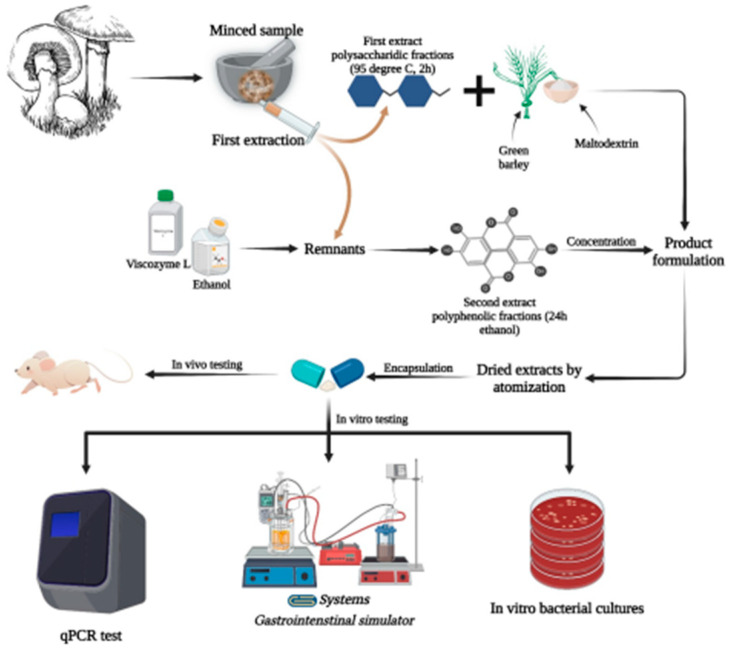
The technological flow for obtaining the extract in atomized form and the characterization options. The figure was created with BioRender App (https://app.biorender.com/; accessed on 22 May 2023).

The extraction was carried out in several phases: the substrate underwent Extraction 1 with water in a ratio of 7.5% (g/100 mL) at 95 °C, for 2 h. Mixture 1 was left to stir on a roller at 100 rpm for up to 24 h without heating for complete extraction of the polysaccharide fraction and small amounts of the phenolic fraction. Extract 1 was obtained by centrifuging the mixture at 4000 rpm for 20 min. The remaining substrate from Extraction 1 was kept for Extraction 2. Extraction 1 was stored in sterile borosilicate glass containers. The recovered substrate was combined with Viscozyme L at 3% (mL/L) and 100% ethanol in a 1:3 weight ratio and subjected to Extraction 2. The mixture was stirred on a roller at 100 revolutions for up to 24 h to fully deplete the substrate and isolate the entire polyphenolic fraction and carotenoid compounds. Extract 2 was obtained by centrifugation at 4000 rpm for 20 min. The substrate, depleted of bioactive compounds, was removed. Extract 2 was pooled with Extract 1 in a 1:3 volume ratio, and the mixture was stored in sterile borosilicate glass containers. The technological flow is shown in Figure 1.

### 2.2. Cytotoxicity of B. edulis Extract

The cytotoxic effect of the extracts was evaluated by measuring the viability of HT-29 cells using the Vybrant^®^ MTT Cell Assay Kit (Thermo Fisher Scientific, Waltham, MA, USA). HT-29 cells at passage 43 were cultured in RPMI 1640 medium (Lonza, Basel, Switzerland) supplemented with 10% FBS (Biochrom, Berlin, Germany) in 96-well plates at 37 °C with 5% CO_2_ until reaching 65% confluence. The medium was removed, and the cells were further incubated for 24 h in a fresh medium containing the extracts at two different concentrations: 10% and 2%. After incubation, the medium was removed, and the cells were washed once with warm PBS and incubated with MTT solution for 2.5 h. The dye was solubilized with DMSO, and the absorbance at 540 nm was read using Synergy HTX (Biotek, Winooski, VT, USA). Cell viability was calculated using the following formula: % survival = (experimental mean absorbance/control mean absorbance) × 100 [12]. In addition to the experimental groups, a control group without any extract was included.

### 2.3. Immune Response Generated by B. edulis Extract

A Ficoll gradient centrifugation protocol was employed to isolate peripheral blood mononuclear cells (PBMCs) from healthy volunteers (*n* = 3). Cell viability was determined using a Thoma cell counting chamber after staining with a 0.4% trypan blue solution. PBMCs at a concentration of 10^5^ cells/mL were resuspended in 90 µL of RPMI 1640 medium (Lonza, Switzerland) supplemented with 10% heat-inactivated FBS, penicillin/streptomycin solution, and 10 µL of the extract was transferred to 96-well plates. The cells were incubated for 20 h at 37 °C with 5% CO_2_. *E. coli* lipopolysaccharides were added at a final 50 ng/mL concentration to stimulate the cells. After incubation, the supernatant was used to quantify interleukin (IL)-1β, -6, and -10 and tumor necrosis factor-alpha (TNF-α) using enzyme-linked immunosorbent assay kits from EIAab (Wuhan, China) according to the manufacturer’s protocols [13,14].

### 2.4. Analysis of Polyphenolic Compounds in Samples by Zonal Capillary Electrophoresis with Diode Array Detector (CE-DAD)

The simultaneous separation of polyphenols (phenolic acids and flavonoids) was carried out on an Agilent G7100 capillary electrophoresis apparatus (Agilent Technologies, Ratingen, Germany) equipped with a diode array detector (DAD). A standard silica capillary with a diameter of 50 µm and an effective length of 63 cm was separated. The background electrolyte (BGE) consisted of a 45 mM sodium tetraborate solution and 0.9 mM sodium dodecyl sulfate, adjusted to a pH = 9.35 (using 1 M HCl). BGE was filtered through 0.2 µm membranes (Millipore, PTFE, Bedford, MA, USA) and degassed before use.

The applied voltage was 25 kV, and the migration temperature was 30 °C. Sample injection was performed hydrodynamically for 10 s at a pressure of 30 mbar. After each migration (45 min), the capillary was washed with 1 M NaOH for 2 min, ultrapure water for 3 min, and BGE for 3 min. The detection of the compounds was carried out in the range of 280–360 nm, and the quantification of the samples was carried out at 280 nm.

The standards used for the analysis were gallic acid, catechin, hesperidin, cinnamic acid, chlorogenic acid, caffeic acid, coumaric acid, ferulic acid, naringenin, rutin, isoquercetin, quercetin, kaempferol, syringic acid, sinapic acid, and myricetin, all of analytical purity > 98% and were purchased from Sigma-Aldrich. The standard stock solutions were prepared in methanol at a concentration of 2 mg mL^−1^. The polyphenolic compounds were identified by comparing the retention times and standard addition. The samples were filtered using membranes with a pore diameter of 0.2 μm (Millipore, Bedford, MA, USA) and degassed before use [15].

### 2.5. In Vitro Simulation of the B. edulis Extract Effects on Dysbiotic Microbiota after Antibiotic Consumption

Following a previously published protocol, the in vitro simulation was conducted using the GIS1 single-chamber simulator [16]. Three microbiotas were used to conduct the in vitro tests and determine the modulation effect after antibiotic administration (amoxicillin and clavulanic acid). These microbiotas were obtained from healthy donors who had not taken any medication in the last six months that could influence the microbiota pattern. These microbiotas were sourced from the ColHumB Collection of the Laboratory of Pharmaceutical Biotechnologies, UASVM Bucharest (www.gissystems.ro). The samples (feces) were handled in accordance with the ethical guidelines of UASVM Bucharest (ColHumB Registration number: 1418/23.11.2017 [11]). Microbiota samples (representing microbial load) were stored in 20% glycerol in the freezer until qPCR analysis. The resulting medium obtained after the simulation was also kept in the freezer for the subsequent analysis of organic acids and antioxidant potential in vitro.

### 2.6. Analysis of the Microbiome by qPCR following The In Vitro Microbial Modulation Process

Total microbial DNA was isolated from 0.8 mL of culture using the PureLink Microbiome DNA Purification Kit (Invitrogen, Waltham, MA, USA). The spectrophotometric method with NanoDrop 8000 (Thermo Fisher Scientific, USA) was used to assess DNA concentration and purity. Power SyberGreen PCR Master Mix 2× (Applied Biosystems, Waltham, MA, USA) and 10 ng of total DNA were introduced into a qPCR reaction with a final volume of 20 µL. Genome copy number quantification of the mainly significant microbial groups in the human gut microbiota was described in the previous study [11,17].

### 2.7. Analysis of Organic Acids Produced following the In Vitro Microbial Modulation Process

A zonal electrophoretic method with reverse polarity was employed to separate short-chain fatty acids (SCFAs) [18]. All used reagents were of analytical purity (purity > 98%): D,L-lactic and butyric acids were purchased from Fluka (Buchs, Switzerland), acetic acid from Riedel-de-Haën (Seelze, Germany), formic, benzoic, succinic, 3-(-4-hydroxyphenyl) lactic, phenyl-lactic, isovaleric and propionic acids were purchased from Sigma-Aldrich (St. Louis, MO, USA). Phosphoric acid 85% and oxalic acid were purchased from Merck (Darmstadt, Germany), cetyltrimethylammonium bromide (CTAB) from Loba Chemie (Fischamend, Austria), and water of chromatographic purity, NaOH 0.1 N and 1 N from Agilent Technologies (Santa Clara, CA, USA).

SCFA separation was performed using an Agilent G7100 capillary electrophoresis apparatus (Agilent Technologies, Ratingen, Germany) with a DAD. A standard silica capillary with a diameter of 50 µm and an effective length of 63 cm was used for the separation. The migration buffer, adjusted to a pH = 6.24, comprised H_3_PO_4_ 0.5 M, (Cetrimonium bromide) CTAB 0.5 mM (pH adjusted with NaOH to 6.24), and 15% methanol. The separation occurred at 25 °C and a voltage of −20 Kv. Samples were injected hydrodynamically for 10 s at 35 mbar. The detection was performed on a DAD at a wavelength of λ = 200 nm.

Between sample migrations, the capillary was washed for 2 min with 1 M NaOH, 2 min with ultrapure water, and 3 min with the BGE. The standard stock solutions were prepared in water and kept at +4 °C. Dilutions of the standard solutions were made daily. The analysis time for each run was 30 min. The separated compounds were identified by comparing the retention times and using standard addition.

Before injection, all the samples were filtered (using the 0.2 μm diameter membranes from Millipore, Bedford, MA, USA) and degassed.

### 2.8. Statistical Analysis

All the parameters were evaluated in triplicate, and the results were expressed as mean ± standard deviation (SD). The statistical analysis was calculated using GraphPad Prism^®^ version 9.5.0 (GraphPad Software, San Diego, CA, USA). The two-way ANOVA was used for cytotoxicity, immune response, and gut microbiota analysis, followed by Dunnett’s test. The significance level for the calculations was set as follows: significant, *p* ≤ 0.05; very significant, *p* ≤ 0.01; highly significant, *p* ≤ 0.001; and extremely significant *p* ≤ 0.0001 using the letters from a to d.

## 3. Results

### 3.1. The Influence of Different Fractions of B. edulis Extract on Cytotoxicity

**Figure 2:**
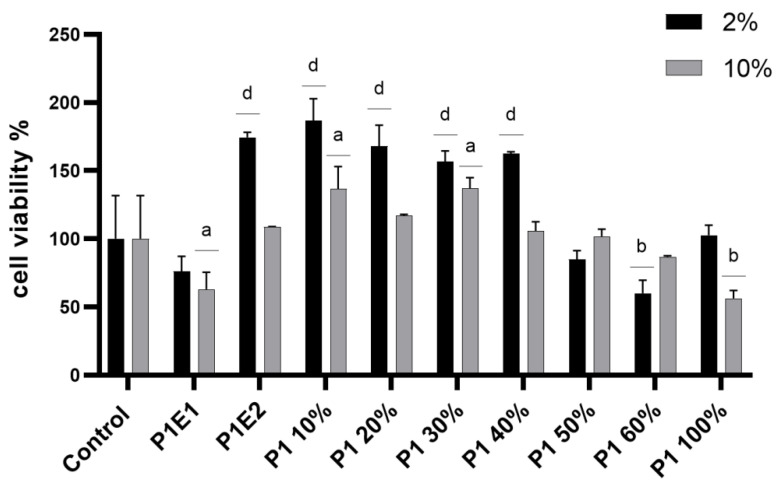
Cytotoxicity of *B. edulis* extracts on HT-29 cell line. Different letters mean statistical differences vs. control, *p* < 0.0001, *n* = 4. Control was represented by HT-29 cells in a solvent. P1E1 means extract after Extraction 1; P1E2 means extract after Extraction 2; P1 10%–P1 100% means final extract mixtures in different concentrations.

Figure 2 illustrates the cytotoxic effect of different concentrations of the extract and the two fractions (polysaccharide—Fraction 1 (P1E1); and polyphenolic—Fraction 2 (P1E2)). A gradual decrease in viability correlated with increasing ethanol concentration was calculated at 2 and 10% of the total sample amount. P1E1 exhibited a more pronounced cytotoxic effect than P1E2 at high concentrations, while P1E2, which contains a polyphenolic fraction, had a proliferative effect at low concentrations.

### 3.2. The Influence of B. edulis Extract Different Fractions on Interleukin Production

**Figure 3:**
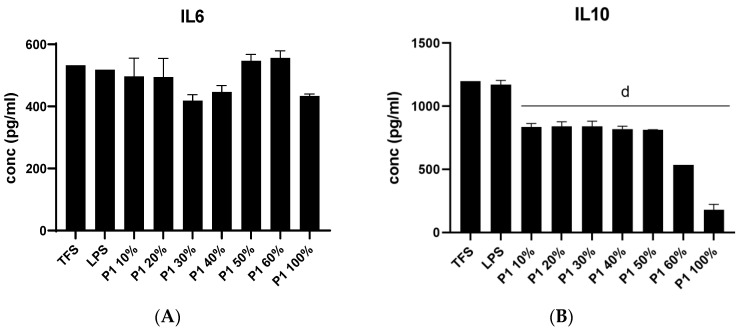

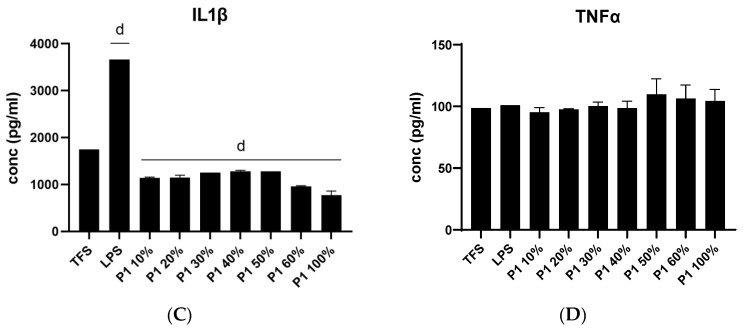
The effect of *B. edulis* extract on the levels of cytokines IL-6 (**A**), IL-10 (**B**), IL-1β (**C**), and TNF-α (**D**) detected by ELISA. TFS—control group; LPS—cells stimulated with lipopolysaccharides of *E. coli*; “d” means statistical difference vs. TFS; *p* < 0.0001; *n* = 3.

Regarding the immune response, the *B. edulis* extract demonstrated (Figure 3) a variable influence on cytokine production. Thus, it reduced the level of IL-10. The inhibitory effect was more pronounced when the extract was solubilized at higher ethanol concentrations [13].

The effect of *B. edulis* extract on pro-inflammatory cytokines TNF-α, IL-6 and IL-1β was variable; it exerted a pronounced inhibitory effect on IL-1β production while not affecting TNF-α and IL-6. The extract solubilized at higher ethanol concentrations inhibited IL-6 and TNF-alpha levels less effectively (*p* ≤ 0.0001).

These results demonstrated that the formula represented a significant anti-inflammatory and immune response mediator [19].

### 3.3. Polyphenolic Pattern of B. edulis Extracts

The phenolic pattern analysis reveals the presence of phenolic acids and flavonoids in the BEE extract; the quantitative analysis (data) of individual bioactive components in the extracts is presented in Table S1. These data are available for individual fractions of the *B. edulis* extract and for the atomized form, similar to the commercial version of the product. The results agreed with previous studies, which also tested other species of mushrooms with medicinal properties [20,21]. In both phases of extraction, the pattern of phenolic compounds was balanced. For example, rutin had an equal proportion in both phases. Also, catechin, naringenin and sinapic acid were isolated, especially in the first extractive phase, demonstrating a specificity towards the extractive process. This detail is important for characterizing a product based on identical mushroom species. These ratios between the groups of identified compounds were also preserved in the atomized product, which preserved the particularities identified in each phase of the extractive process.

### 3.4. Modulation through the B. edulis Extract Effects on Dysbiotic Microbiota after Antibiotic Consumption

**Figure 4:**
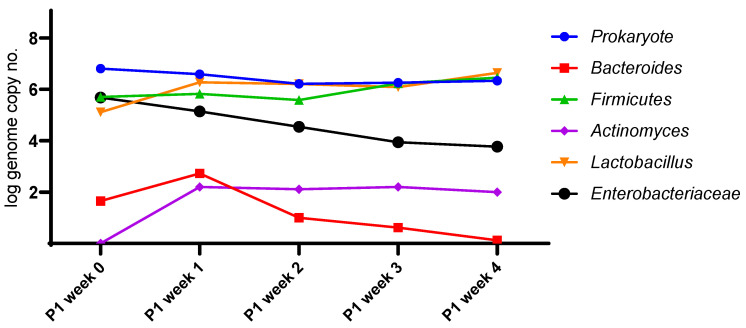
Evolution of the microbial pattern after administration of *B. edulis* extract, *n* = 3.

**Figure 5:**
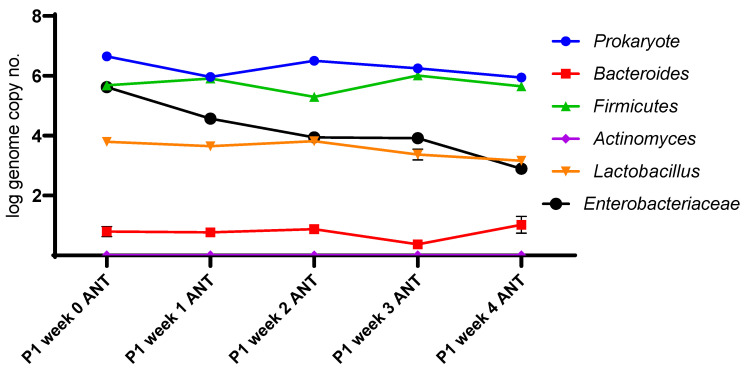
Evolution of the microbial pattern of antibiotic-treated microbiota after administration of *B. edulis* extract, *n* = 3.

Regardless of prior antibiotic use, administering the extract to the microbiota successfully modulated the microbiota (Figure 4 and Figure 5). The microbiota pattern was stabilized after the first weeks following the extract’s in vitro administration. This phenomenon was directly correlated with pH evolution. Notably, the extract reduced the presence of *Enterobacteriaceae*, some species being recognized as biomarkers in type 2 diabetes [22].

*Enterobacteriaceae* populations decreased by approximately 2 log/mL within the first three weeks of fermentation. Simultaneously, the quantities of the *Lactobacillus* and *Firmicutes* strains equally increased by 1.5 log/mL, leading to a general stabilization of the fermentation conditions. The dynamics of these two groups were similar during monitoring, with lactobacilli emerging as the most dominant members of the *Firmicutes* family. Beyond the third week, the pH values stabilized, reflecting a direct correlation with the metabolomic pattern. The *Bacteroides* group decreased by 1.5 log/mL during fermentation, while *Actinomyces* increased by 2 log/mL, with group shifts observed within the first week (Figure 4).

The main microbial groups in human microbiota did not vary during the 4 weeks following antibiotic treatment, except for a roughly 2 log/mL decrease in *Enterobacteriaceae.* This effect was also observed in samples not exposed to antibiotics. Small variations were observed in *Bacteroides*, lactobacilli, and *Firmicutes* during the 4 weeks, remaining around the same initial copy numbers at the end. The members of actinomycetes were not detected during the stimulation process, while lactobacilli were less prevalent in the antibiotic-treated microbiota.

The microbiota and host relationship influences the metabolomic pattern, as shown in Table S2. Propionic and butyric acids had high values in the samples not treated with antibiotics and after two weeks of antibiotic therapy. Isovaleric acid was abundant, demonstrating increased bacterial diversity and intense metabolomic activity. The high presence of these acids, acting as biomarkers, exhibited modulatory capacity in antibiotic-induced dysbiosis. These acids, essential for maintaining homeostasis, had an increasing trend. Notably, propionic acid exhibited accelerated accumulation during the recovery from dysbiosis, being identified in the multiplication phase of the microbiota and demonstrating a correlation with the bacterial exponential growth phase. These results showed that bacterial multiplication, rather than the test products directly, influenced metabolomic stimulation. Acetic and lactic acids were found in high concentrations, demonstrating a strong inhibitory effect against pathogenic strains.

## 4. Discussion

The most important compounds identified in mushroom extracts are polysaccharides represented by β-(1,3)/(1,6)-d-glucans. Polysaccharides can influence nutritional outcomes based on their structure and function, and are considered functional compounds. They are used to customize or modify nutrition to achieve specific health goals. The association of polysaccharides with other compounds with a functional role (phenolic compounds) makes it possible to include these products (or extracts containing them) in the category of natural nutraceuticals [23]. Numerous studies have shown that the interaction between polyphenols and carbohydrates determines a series of positive effects in the large intestine. The association between carbohydrates (in our case, polysaccharides from *Boletus edulis*) and polyphenols (catechin, rutin, naringenin etc.) can lead to an increase in the bioavailability and antioxidant activity of the latter or the appearance of some metabolites resulting from their degradation with positive effects on gut processes [24,25,26]. The results obtained from BEE testing suggest that the extracted compounds synergistically restore balance in dysbiotic microbiota.

The immune response exerted by *B. edulis* extracts demonstrates specificity towards different types of cytokines. The ability of these extracts to reduce certain types of pro-inflammatory cytokines indicates their potential as an adjuvant in the classical therapy of inflammatory diseases. The reduced cytotoxicity and pronounced inhibition of certain pro-inflammatory cytokines demonstrated a link between inflammation progression and the development of certain types of tumors. The latter is attributed to oxidative stress, and the development of functional supplements to complement conventional therapies is important in reducing the incidence of tumors. In the case of the IL-10 cytokine, new data have shown that its inhibition is important in reducing pro-inflammatory conditions. In humans, increases in the levels of IL-1β and TNF-α are associated with symptoms such as fever, inflammation, and tissue damage. Various therapeutical agents, such as antibodies or receptor antagonists, have been developed to block the activity of specific cytokines, reducing inflammatory processes in patients with inflammatory bowel disease or rheumatoid arthritis. *B. edulis* extract, by influencing immune response, can model immune response in some inflammatory diseases.

The cytotoxic effect and the selective impact on the tested cytokines may lead to the development of effective adjuvants in cancer therapy [27]. Also, by administering the extract, we aim to mitigate antibiotic-induced dysbiosis-associated oxidative stress by decreasing some inflammatory processes that can prevent tumor development in the human colon. Naturally, recovering from dysbiosis is highly desirable as it eliminates the cause determining the inflammatory process.

The associated effect of the extract—the microbial modulation and inhibition of pro-inflammatory cytokines—represents an important study result. Cytokine responses depend on the microbial host’s stimulus [28]. IL-6 and IL-1β are responsible for the acute phases, depending on the genes present in the microbiota pattern. This observation was reinforced by differing levels of the two cytokines, demonstrating a modulation achieved by the functional extract of *B. edulis* [29].

This study evaluated the role of *Boletus edulis* extract in mitigating antibiotic-associated dysbiosis. Also, the metabolomic pattern associated with the cytotoxic effect was established. In addition, the study demonstrated a direct relationship between microbiota modulation and the anti-inflammatory response. As in previous studies, the exact mechanisms underpinning these biological effects remain poorly understood and are beyond the explanatory power of an in vitro study [30]. An important role could be attributed to pH, a biomarker showing the stabilizing effect of the extract’s administration on the modulation process. The high concentration of organic acids and their diverse pattern demonstrated a high bacterial diversity that ensured an increase in the plasticity of the intestinal microbiome [31].

According to recent studies [32], a diversified pattern of SCFAs (Table S2) demonstrated a cytotoxic effect based on modulating the immune response. The stimulation of some key anti-inflammatory cytokines supports these results, demonstrating the biological value of *Boletus edulis* extract.

Various bioactive compounds may offer health benefits. These compounds contribute to its potential nutritional and therapeutic value. Boletus edulis, a new atomized extract, is rich in antioxidants such as phenolic compounds, flavonoids, and polysaccharides. These antioxidants help protect cells from oxidative damage caused by free radicals, reducing the risk of chronic disease and promoting overall health. The obtained data align with past studies that suggest that *B. edulis* extract may possess immunomodulatory effects. The polysaccharides present (which are yet undetermined data) in the extract may stimulate the immune system, enhancing its response to infections and supporting its overall immune function. Certain compounds found in *B. edulis* extract have demonstrated anti-inflammatory properties. These components may help reduce inflammation and potentially alleviate symptoms associated with inflammatory conditions. *B. edulis* is a nutrient-rich mushroom containing essential vitamins, minerals, and dietary fiber. While the extract retains the nutritional profile of the whole mushroom, it may still provide effects based on these beneficial nutrients. It is important to note that the biological value and specific health benefits of our Boletus edulis extract may vary depending on factors such as extraction parameters, processing, and dosage. Individual responses to the extract may also vary, specific to different physiological conditions.

Mushrooms are generally considered safe to consume and have no known direct interactions with antibiotics. Antibiotics can disrupt the balance of beneficial bacteria in the colon (leading to issues such as diarrhea or an increased risk of fungal infections). As a natural functional source, the obtained extract contains prebiotic fibers that may support the growth of beneficial gut bacteria. The administration of *B. edulis* extract and antibiotics promotes a healthy gut microbiome fingerprint and well-being.

The correlation was established in vitro through cytotoxicity analysis, suggesting that the administration of the extract can have a modulatory role and a protective effect against colon cancer. Antibiotic-induced dysbiosis increases the risk of developing colon cancer [33]. Our results successfully demonstrated the role of metabolomic modulation as a therapeutic target in controlling immune response and cytokine levels in target populations. In addition, the study also demonstrated the bioavailability of the bioactive compounds manifested by ameliorating some dysbiosis-related side effects at the microbiota level.

In conclusion, our comparative results demonstrated that the administration of the extract has a modulatory effect associated with compositional normalization. The diversity of the intestinal microbiome increased, and the metabolomic pattern was characterized by an increased concentration of organic acids acting as biomarkers. Future analyses are needed to demonstrate that the effect is sustained in the case of other drugs and pathologies that affect gut microbial diversity, limit the metabolomic pattern, or reduce anti-inflammatory capacity, affecting homeostasis.

## Data Availability

Not applicable.

## Supplementary Materials

The following supporting information can be downloaded at https://www.mdpi.com/article/10.3390/life13071481/s1: Table S1. The pattern of phenolic compounds in the *B. edulis* extracts; Table S2. Concentrations of short-chain fatty acids (SCFAs) after in vitro simulation.
