# Supplementary material for: Boletus edulis Extract—A New Modulator of Dysbiotic Microbiota
Source: Life (Basel). 2023 Jun 30;13(7):1481. doi: 10.3390/life13071481 (PMC10381576; doi:10.3390/life13071481)
Supplement: Supplementary file 1 [file life-13-01481-s001.zip › Table S1_rev.pdf]

Table S1. The pattern of phenolic compounds in the *B. edulis* extracts

No	Compounds	P1F1 (μg mL^{-1})	P1F2 (μg mL^{-1})	P1Fin (μg DW^{-1})*
1	Hesperidin	10.561±0.771	6.812±0.340	17.967±0.621
2	Catechin	55.556±1.136	28.643±0.618	83.940±0.137
3	Naringenin	13.773±0.66	5.649±0.186	17.077±0.174
4	Rutin	152.619±3.633	157.794±3.443	311.476±3.444
5	Cinnamic acid	3.326±0.107	3.622±0.109	6.829±0.057
6	Chlorogenic acid	4.590±0.088	3.495±0.146	9.239±0.167
7	Sinapic acid	15.05±0.435	5.308±0.026	22.798±0.111
8	Syringic acid	5.751±0.094	3.879±0.107	8.101±0.101
9	Ferulic acid	2.778±0.084	4.376±0.096	7.218±0.044
10	Myricetin	1.397±0.093	2.526±0.078	3.392±0.016

* - DW-dry weight after atomization
