# Supplementary material for: Boletus edulis Extract—A New Modulator of Dysbiotic Microbiota
Source: Life (Basel). 2023 Jun 30;13(7):1481. doi: 10.3390/life13071481 (PMC10381576; doi:10.3390/life13071481)
Supplement: Supplementary file 1 [file life-13-01481-s001.zip › Table S2_1rev.pdf]

Table S2. Concentrations of short-chain fatty acids in bacterial culture samples

Sample	Formic acid g L^{-1}	Oxalic acid g L^{-1}	Succinic acid g L^{-1}	Acetic acid g L^{-1}	Propionic acid g L^{-1}	Lactic acid g L^{-1}	Butyric acid g L^{-1}	Benzoic acid g L^{-1}	Izovaleric acid g L^{-1}	Phenyl lactic acid g L^{-1}	3-(4-hydroxyphenyl) lactic acid g L^{-1}
**M**	0.158±0.028	0.005±0.00	0.087±0.006	0.292±0.016	0.377±0.031	0.889±0.003	0.060±0.001	1.71±0.10	0.061±0.002	0.004±0.00	0.002±0.00
**W1**	-	0.004±0.00	0.256±0.002	0.767±0.010	0.017±0.001	5.291±0.086	0.886±0.024	1.71±0.05	0.370±0.030	0.007±0.00	0.003±0.00
**W2**	-	-	0.175±0.002	0.832±0.025	0.050±0.01	5.960±0.100	0.060±0.011	1.60±0.07	0.306±0.002	0.006±0.00	0.003±00
**W3**	0.062±0.002	0.006±0.00	0.310±0.003	2.131±0.019	0.042±0.006	11.348±0.106	1.524±0.012	1.34±0.10	0.567±0.030	0.010±0.001	0.006±0.00
**W4**	0.064±0.003	0.007±0.00	0.286±0.007	2.044±0.010	0.026±0.001	16.114±0.384	0.105±0.003	7.87±0.15	0.562±0.030	0.010±0.001	0.007±0.00
**Ma**	0.111±0.023	-	0.181±0.004	0.344±0.05	0.061±0.00	0.745±0.040	0.039±0.002	-	0.272±0.012	0.010±0.000	-
**AntibP1**	0.253±0.038	0.053±0.006	0.522±0.014	0.763±0.03	0.823±0.018	2.129±0. 060	0.344±0.013	0.501±0.005	2.408±0.130	-	0.077±0.006
**W1**	0.488±0.037	0.111±0.001	0.493±0.012	1.103±0.043	0.491±0.015	1.069±0.014	0.548±0.035	0.735±0.010	2.678±0.110	0.154±0.000	0.022±0.000
**W2**	0.422±0.039	0.100±0.002	0.259±0.014	1.190±0.036	0.346±0.003	1.423±0.056	1.179±0.061	0.417±0.001	1.583±0.031	0.110±0.004	0.020±0.000
**W3**	-	0.009±0.00	0.354±0.003	0.744±0.052	0.162±0.00	2.270±0.029	0.495±0.04	-	0.688±0.020	-	0.052±0.001
**W4**	-	0.006±0.00	0.306±0.012	0.584±0.026	0.179±0.018	2.001±0.020	0.518±0.006	-	0.601±0.010	-	0.011±0.000
